# Incidence of Metabolic Risk Factors Among Healthy Obese Adults: 20-Year Follow-Up

**DOI:** 10.1016/j.jacc.2015.06.014

**Published:** 2015-08-18

**Authors:** Joshua A. Bell, Mark Hamer, G. David Batty, Archana Singh-Manoux, Séverine Sabia, Mika Kivimäki

There is growing evidence that obese adults without metabolic risk factor clustering (the so-called “healthy obese”) progress to unhealthy obesity over time (1). However, the pathophysiological changes underlying the long-term transition into an unhealthy obese state have not been well characterized. To inform clinical management of healthy obesity, we aimed to identify the metabolic risk factors responsible for this transition, as well as the timing of their onset.

Repeat clinical data were drawn from the Whitehall II cohort study of British adults. We grouped participants as normal-weight (body mass index [BMI] 18.5 to 24.9 kg/m^2^), overweight (BMI 25 to 29 kg/m^2^), or obese (BMI ≥30 kg/m^2^), and as healthy (2) if they were free of any the following characteristics: high-density lipoprotein cholesterol <1.03 mmol/l (men) and <1.29 mmol/l (women); blood pressure ≥130/85 mm Hg or antihypertension medication use; fasting plasma glucose ≥5.6 mmol/l or diabetic medication use; triglycerides ≥1.7 mmol/l; and homeostatic model–assessed insulin resistance >2.83 (baseline 90th percentile value). Participants provided written informed consent. The University College London research ethics committee provided ethical approval.

Cross-tabulations were used to describe incidence of each of these 5 metabolic risk factors at 5- (1997 to 1999), 10- (2002 to 2004), 15- (2007 to 2009), and 20-year (2012 to 2014) follow-ups for healthy normal-weight, overweight, or obese participants at baseline (1992 to 1994). Poisson regression models with robust error variances were used to estimate age-, sex-, and ethnicity-adjusted incidence ratios and 95% confidence intervals for having each metabolic risk factor at follow-up for healthy obese compared with healthy normal-weight adults at baseline.

Among 2,878 adults with anthropometric and metabolic risk factor data at each time point, 1,120 adults (39 to 61 years of age; 68% male) were free of all metabolic risk factors at baseline. This initially healthy status was progressively rarer among those in higher BMI groups, representing 51.5%, 25.8%, and 13.4% of normal-weight, overweight, and obese adults, respectively. Of the healthy obese participants, 57.1% had at least 1 metabolic risk factor at the 5-year follow-up, with corresponding proportions being 64.3% at 10 years and 78.6% at 20 years. These proportions were smaller (32.8%, 46.7%, and 60.3%) among initially healthy normal-weight participants.

After 5 years (Figure 1), relative to initially healthy normal-weight adults, initially healthy obese adults were 4.40 times more likely to be insulin resistant, 3.35 times more likely to have high blood glucose, and 1.92 times more likely to be hypertensive (all p < 0.05). Incident insulin resistance remained higher over all subsequent follow-ups among baseline healthy obese compared with healthy normal-weight adults. Case numbers were small for 20-year incidence of low high-density lipoprotein cholesterol (2 cases) and high triglycerides (3 cases) among healthy obese adults, with little difference in risk compared with healthy normal-weight adults over time. Additional data are available on request.

The risk of developing insulin resistance, high blood glucose, and hypertension was 2 to 5 times higher among initially healthy obese adults compared with their healthy normal-weight counterparts, and these changes were evident after only 5 years of follow-up. There was little difference in progression to dyslipidemia. However, the key factor explaining the long-term decline of healthy obesity was insulin resistance, which was consistently most common among healthy obese adults over time.

Healthy obese adults are known to experience an elevated future risk of type 2 diabetes (3) and cardiovascular disease (4) compared with healthy normal-weight counterparts. That insulin resistance is an established indicator of future impaired glucose metabolism (5) may explain their much higher incidence of type 2 diabetes (relative risk near 4.0) (3) and slightly higher incidence of cardiovascular disease (relative risk near 1.2) (4), given that earlier onset of risk factors leads to a greater cumulated exposure and higher disease risk. Overall, our findings suggest that healthy obesity is strongly linked with future insulin resistance that subsequently induces cardiometabolic pathology.

## Figures and Tables

**Figure 1:**
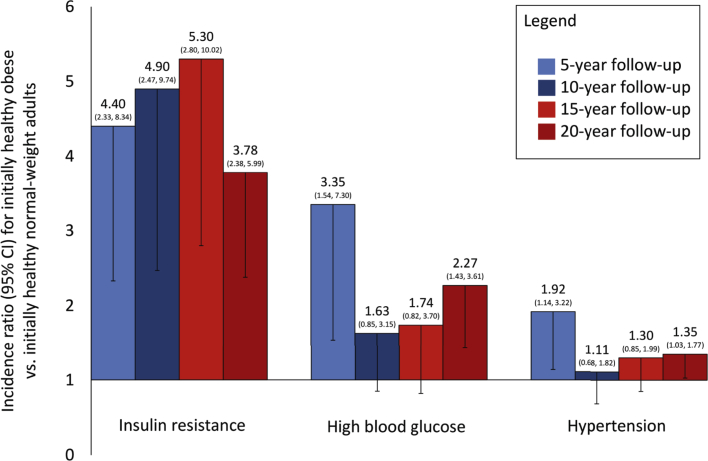
Incidence of Metabolic Risk Factors Among Initially Healthy Obese Compared With Initially Healthy Normal-Weight Adults Over 20 Years (n = 1,120) Results are incidence ratios and 95% confidence intervals (CI) for having each metabolic risk factor at follow-up, on the basis of Poisson regression models with robust error variances. Models are adjusted for age, sex, and ethnicity. Little difference in high-density lipoprotein cholesterol or triglycerides was observed between groups. Baseline healthy status is defined as having none of 5 metabolic risk factors (hypertension, low high-density lipoprotein cholesterol, high triglycerides, insulin resistance, and high blood glucose).
